# Multimorbidity Profiles and Infection Severity in COVID-19 Population Using Network Analysis in the Andalusian Health Population Database

**DOI:** 10.3390/ijerph19073808

**Published:** 2022-03-23

**Authors:** Jonás Carmona-Pírez, Ignatios Ioakeim-Skoufa, Antonio Gimeno-Miguel, Beatriz Poblador-Plou, Francisca González-Rubio, Dolores Muñoyerro-Muñiz, Juliana Rodríguez-Herrera, Juan Antonio Goicoechea-Salazar, Alexandra Prados-Torres, Román Villegas-Portero

**Affiliations:** 1 EpiChron Research Group, Aragon Health Sciences Institute (IACS), IIS Aragón, Miguel Servet University Hospital, 50009 Zaragoza, Spain; ignatios.ioakeimskoufa@fhi.no (I.I.-S.); agimenomi.iacs@aragon.es (A.G.-M.); bpoblador.iacs@aragon.es (B.P.-P.); franciscagonzalezrubio@gmail.com (F.G.-R.); sprados.iacs@aragon.es (A.P.-T.); 2 Health Services Research on Chronic Patients Network (REDISSEC), ISCIII, 28029 Madrid, Spain; 3 Delicias-Sur Primary Care Health Centre, Aragon Health Service (SALUD), 50009 Zaragoza, Spain; 4 Red de Investigación en Cronicidad, Atención Primaria y Promoción de la Salud (RICAPPS), ISCIII, 28029 Madrid, Spain; 5 WHO Collaborating Centre for Drug Statistics Methodology, Norwegian Institute of Public Health, NO-0213 Oslo, Norway; 6 Department of Drug Statistics, Division of Health Data and Digitalisation, Norwegian Institute of Public Health, NO-0213 Oslo, Norway; 7 Drug Utilization Work Group, Spanish Society of Family and Community Medicine (SEMFYC), 08009 Barcelona, Spain; 8 Subdirección Técnica Asesora de Gestión de la Información, Servicio Andaluz de Salud (SAS), 41071 Seville, Spain; dolores.munoyerro.sspa@juntadeandalucia.es (D.M.-M.); juliana.rodriguez.sspa@juntadeandalucia.es (J.R.-H.); jantonio.goicoechea@juntadeandalucia.es (J.A.G.-S.); romanp.villegas@juntadeandalucia.es (R.V.-P.)

**Keywords:** COVID-19, multimorbidity, chronic diseases, network analysis, severity, disease burden

## Abstract

Identifying the population at risk of COVID-19 infection severity is a priority for clinicians and health systems. Most studies to date have only focused on the effect of specific disorders on infection severity, without considering that patients usually present multiple chronic diseases and that these conditions tend to group together in the form of multimorbidity patterns. In this large-scale epidemiological study, including primary and hospital care information of 166,242 patients with confirmed COVID-19 infection from the Spanish region of Andalusia, we applied network analysis to identify multimorbidity profiles and analyze their impact on the risk of hospitalization and mortality. Our results showed that multimorbidity was a risk factor for COVID-19 severity and that this risk increased with the morbidity burden. Individuals with advanced cardio-metabolic profiles frequently presented the highest infection severity risk in both sexes. The pattern with the highest severity associated in men was present in almost 28.7% of those aged ≥ 80 years and included associations between cardiovascular, respiratory, and metabolic diseases; age-adjusted odds ratio (OR) 95% confidence interval (1.71 (1.44–2.02)). In women, similar patterns were also associated the most with infection severity, in 7% of 65–79-year-olds (1.44 (1.34–1.54)) and in 29% of ≥80-year-olds (1.35 (1.18–1.53)). Patients with mental health patterns also showed one of the highest risks of COVID-19 severity, especially in women. These findings strongly recommend the implementation of personalized approaches to patients with multimorbidity and SARS-CoV-2 infection, especially in the population with high morbidity burden.

## 1. Introduction

Coronavirus disease 2019 (COVID-19), caused by Severe Acute Respiratory Syndrome Coronavirus 2 (SARS-CoV-2), was declared a Public Health Emergency of International Concern approximately one month after the report of the first cases, with 171 deaths at that time [1] and representing one of the biggest public health challenges in the last decades. Today this figure exceeds 5.5 million, with the total number of global deaths attributable to the COVID-19 pandemic estimated to be substantially higher with even higher the incidence of hospitalization [1,2,3]. During the evolution of the pandemics, numerous SARS-CoV-2 variants have been identified, some of them with possible increased transmissibility, risk of reinfection, hospitalization, and mortality compared with the wild type [1,4,5]. The rapidly changing environment (virus variants, vaccination rates, health policies, interventions, and so forth) puts high pressure on public healthcare systems, prioritizing identifying the population at risk of COVID-19 severity constantly, especially in terms of hospitalization and mortality.

Since the first reported cases, it has been known that advanced age is one of the most critical factors associated with a higher risk of negative outcomes, pointing out the urgent need to implement preventive strategies in the elderly population [6,7,8,9,10,11]. However, people of all ages are susceptible to developing severe COVID-19, highlighting the importance of identifying risk factors considering age and sex. Numerous studies have searched for associations between specific chronic diseases and poorer prognosis, mainly mortality. Concurrent cardiovascular diseases, diabetes, obstructive pulmonary diseases, chronic renal failure, and neoplasms were found to be risk factors for COVID-19 hospitalization and mortality [12,13,14]. These findings are mainly observational works that estimated associations for each condition alone, with the total number of chronic conditions varying considerably between the different studies. However, the coexistence of two or more chronic diseases, also known as multimorbidity, is very common in the general population [15]. Especially amongst the elderly, it is more likely for an individual to have two or more chronic conditions than only one single disease [16,17]. The presence of multimorbidity can also imply disease–disease interactions that could substantially impact clinical outcomes. Some discrepancies in the literature on whether a particular disease is a risk factor of COVID-19 severity could be partly attributed to a variety of interactions in a person with multiple chronic conditions, including disease–disease, disease–drug, and of course, drug–drug interactions. Thus, a more precise estimation of the clinical profiles associated with a higher risk of COVID-19 severity should consider multimorbidity.

It seems logical to expect that some combinations of diseases would have a higher severity risk than others. Studying all possible combinations of diseases could be a practically hard or even impossible task, especially when including an extensive list of chronic conditions. On the other hand, it is well-known that there are systematic associations between chronic conditions; chronic diseases do not appear randomly, but they tend to cluster in groups [18]. Various sophisticated methods have been applied in practice to identify multimorbidity patterns, such as factor analysis, cluster analysis, and network analysis [18,19,20,21]. These methods allow us to make a reasonable estimation of all different clinical profiles that describe most of the population with multimorbidity, sometimes with clinically unexpected combinations of chronic diseases. Then, we can assess the risk of COVID-19 severity for each of these clinical profiles, providing a more realistic, person-centered approach—this is a public health research priority.

Network analysis is a powerful tool to detect networks of individuals and chronic conditions through correlations amongst them, [20,22,23] with high applicability in epidemiology and public health to unlock the potential of real-world data for health research, primarily when used in large-scale epidemiologic studies [24]. This methodology was previously used in a study analyzing COVID-19 patient networks and their infection severity predictors, but considering only patients’ diagnoses recorded in the primary care setting [23]. By applying network analysis in real-world data, this study explores multimorbidity profiles in the whole population with laboratory-confirmed SARS-CoV-2 infection in the Spanish region of Andalusia and estimates each patient network’s likelihood of hospitalization and mortality considering age, sex, and the whole spectrum of patients’ chronic diseases from both primary and hospital care.

## 2. Materials and Methods

### 2.1. Design and Study Population

We performed an observational, retrospective study in the Andalusian Health Population Database (BPS) [25], which includes demographic and clinical information of all the users of the public health system in Andalusia, a region of southern Spain with a reference population of 8.5 million inhabitants. The clinical diagnoses in the BPS were coded by clinical documentalists for the hospitalization, major outpatient surgery, and day care episodes, and by an automatic self-developed encoder for hospital emergencies and electronic health records (EHRs) from primary care. Accumulated data included 13.8 million people who were registered in the database from 2001 to 2021, with a total of 601 million diagnoses, 473 million of them from primary care. Andalusian Health System provides universal and free health coverage for all citizens and is used by approximately 99% of the reference population in the region [25]. For this study, we included all 166,242 individuals aged 15 years or older with laboratory-confirmed SARS-CoV-2 infection and at least one chronic condition from 15 June 2020 to 19 December 2020.

This study aimed to (1) identify profiles of COVID-19 patients according to their baseline morbidity using network analysis, (2) to characterize the multimorbidity patterns obtained, and (3) to analyze their impact on infection severity in comparison to chronic patients without multimorbidity.

The Clinical Research Ethics Committee of Andalusia (CCEIBA) approved the research protocol for this study (2309-N-21). CCEIBA waived the requirement to obtain informed consent from the patients included in this study due to the use of anonymized data and the epidemiological nature of the project. We performed this study following the Spanish Law on the protection of personal data (LOPD 15/1999 of December 14) and the Declaration of Helsinki. 

### 2.2. Study Variables and Data Sources

For each person, we analyzed sex, age (stratified in three groups: 15–64, 65–79, ≥80 years), and all chronic baseline conditions from patients’ EHRs present at the time of inclusion in the study. Diagnoses were classified using the International Classification of Diseases, 9th and 10th revision, and then these codes were assigned to 226 clinical categories using the Clinical Classification Software [26]. A total of 153 of these categories were classified as chronic using the Chronic Condition Indicator software [27], which considers as chronic those conditions present at least during the last 12 months and that meet one or both of the following criteria: (a) require continuous interventions using medical products, special equipment, and services; and (b) entail limitations on self-care, social interactions, and independent living. Clinicians in the group renamed some diagnostics to facilitate their clinical interpretation. Multimorbidity was defined as the presence in a patient of at least two chronic diseases meeting the previous criteria.

We analyzed patient mortality and hospitalization during the follow-up. To analyze mortality, we followed patients for a maximum of 30 days, as we considered it difficult to attribute to COVID-19 infection an event occurring after this period, considering the exact cause of death was not available. Regarding hospitalization (including admission to the ICU), we considered a consequence of the infection those occurring within 15 days of the index day. We also considered hospitalization occurring up to 15 days before the index day since some patients were diagnosed after hospitalization. The study variables were obtained from patients’ EHRs and from the users’ health databases.

### 2.3. Statistical Analysis

First, we described the demographic and clinical characteristics of the study population as frequencies or means accompanied by their respective standard deviations. 

Then, we applied network analysis in the population with multimorbidity in each sex and age interval subgroup to identify multimorbidity profiles (i.e., groups of similar patients based on all their chronic baseline conditions). Network science analyzes graphs of linked components to study complex systems, tackling the challenge to infer their collective behavior based on the interconnection of the system’s elements [28]. In this study, the linked components were the patients, which were interconnected based on their clinical similarities in the form of networks.

We used the Jaccard index (JI) to measure the similarity between patients due to the binary nature of the diagnostic variables (i.e., absence/presence), as performed in previous studies on multimorbidity patterns [19,23,29]. This index measures the similarity between patients based on individual and shared chronic diseases and ignores clinical diagnostics that none of them has. The JI formula is the intersection over the union of the sets studied, and we applied it to each pair of patients analyzing their chronic disease datasets. When two individuals are identical, their JI = 1, and if they share no values, their JI = 0. A link between two given patients was created if the JI between them was ≥0.33 to include patients who share half or more of their chronic diseases with another patient. Thus, each node represents a different patient, and a link means a JI ≥ 0.33 between patients, as already applied in previous work [23], combining clinical and statistical criteria for building the patient networks. This cutoff allowed the inclusion of almost all the patients with multimorbidity (144,990 out of 145,070 patients). At the same time, it only included 3.27% of all possible combinations between patients (111,371,820 out of 3,404,243,854 of all possible combinations), which saved computation memory.

We used the network’s modularity to search for communities of patients within each network, as previously used in comorbidity and multimorbidity pattern studies [20,22,23,30]. We used the Leiden algorithm to guarantee well-connected communities [31]. Modularity measures the density of links inside communities compared to the links between them [32]. We detected communities, also called clusters of patients, by optimizing modularity in an iterated procedure until modularity ceased to increase [33]. Community detection methods, such as the Leiden or Louvain algorithms, allow the number and size of the clusters to be determined by the network’s structure [34] and not by the researcher. We assigned each individual to a community or group of patients through this step.

Once the clusters of patients were identified for each subpopulation, and with the aim to characterize multimorbidity patterns obtained, the prevalence of each chronic condition was calculated. We also measured their observed/expected (O/E) prevalence ratio (i.e., the disease prevalence observed in a specific cluster divided by the observed disease prevalence in the stratum of reference). We included a chronic condition in a pattern if (1) the disease prevalence was ≥25%, or (2) the O/E prevalence ratio was ≥2 [35,36], and the disease prevalence was ≥1%. Then, all clinicians named the patterns by consensus, considering the most relevant diseases within each profile according to their disease prevalence and O/E prevalence ratio, and in line with the names given in the literature.

Finally, to calculate the impact on infection severity of each multimorbidity profile, we obtained age-adjusted logistic regression models in each subpopulation. We used the group of patients with one chronic condition as the reference group, and we calculated age-adjusted odds ratios (ORs) accompanied by their respective 95% confidence intervals (CI), which represented the probability of death and hospitalization for each multimorbidity profile compared with having only one chronic disease.

We performed all the analyses using RStudio software (Version 1.4.1106, RStudio, Boston, MA, USA).

## 3. Results

From 15 June 2020 to 19 December 2020, 166,242 cases (56.1% women) of laboratory-confirmed SARS-CoV-2 infection were reported in the Autonomous Community of Andalusia in Spain. The infected individuals’ mean age was 47.4 ± 19.6 standard deviation (SD). Approximately eight in ten men and nine in ten women had multiple chronic conditions, with an average of five chronic diseases per patient. Almost all of the elderly population 65 years of age and older (98.6%) had multimorbidity. One in ten SARS-CoV-2 infected individuals presented a severe infection in terms of hospitalization and mortality; this figure was three times higher in the elderly and four times higher in the population 80 years of age and older. Table 1 presents a general overview of the study population.

Network analysis revealed the existence of different multimorbidity profiles between sexes and age groups. Table 2 presents the prevalence of each multimorbidity profile and its corresponding age-adjusted odds ratio of infection severity compared with chronic patients without multimorbidity. More detailed information regarding the clinical component of each network is given in the Supplementary Material.

### 3.1. Multimorbidity Profiles in Men

We observed seven multimorbidity profiles in young and middle-aged men. In the sensory organ disorder pattern, almost all men had a sense organ disorder, and one in three also had respiratory comorbidity. An allergic component was a common finding in this network. In the eye disorders-respiratory group, almost all individuals had eye disorders and respiratory conditions, with four in ten men also having asthma. A similar profile was the respiratory pattern, with a high prevalence of respiratory disorders and half of the individuals also having asthma. Mental health problems were the main comorbidity in the mental health group, with a diagnosis of anxiety in nine in ten men of this cluster. The neurologic-mental health pattern had a similar clinical profile, with the coexistence of mental health comorbidity, neurologic, and developmental disorders. Dyslipidemia and arterial hypertension were amongst the most common conditions in men 15–64 years of age; three in ten individuals had a lipid metabolism disorder, and one in four had arterial hypertension. Almost all individuals of the dyslipidemia pattern had lipid metabolism disorders. In the cardiovascular-metabolic pattern, dyslipidemia, hypertension, obesity, and other cardiovascular and metabolic conditions were in coexistence with a huge variety of other comorbidities, such as obstructive sleep apnea, osteoarthritis, and kidney diseases. This clinical profile was the most prevalent and with the highest likelihood of COVID-19 severity in men 15–64 years of age (13.7% higher); individuals of the cardiovascular-metabolic pattern were more likely to present a severe SARS-CoV-2 infection (in terms of hospitalization and mortality) in comparison with infected individuals of the same age group without multimorbidity. 

We observed four multimorbidity patterns in the elderly population 65–79 years of age. Arterial hypertension, dyslipidemia, and diabetes mellitus are present in all multimorbidity profiles in this age group. We noticed that differences in the prevalence of these conditions and additional comorbidity determine the likelihood of hospitalization and mortality. The metabolic pattern consists of arterial hypertension, dyslipidemia, and diabetes mellitus and has a 15.6% higher likelihood of severity. When a man with prostatic hyperplasia has at least one of these metabolic conditions (a clinical profile of the prostatic-metabolic pattern), the likelihood is 19.9%. The musculoskeletal-metabolic pattern describes the population with osteoarthritis, back problems, and metabolic conditions. The most prevalent multimorbidity profile was the coexistence of cardiovascular diseases, respiratory disorders, metabolic conditions, and additional comorbidity; this network had the highest likelihood of hospitalization and mortality (42.9% higher than the control group).

In the oldest-old population, we observed four multimorbidity patterns. In addition to arterial hypertension, dyslipidemia, and diabetes mellitus, three more conditions are present in all multimorbidity profiles: prostatic hyperplasia, neoplasia, and cataracts. Their coexistence has been associated with a higher likelihood of COVID-19 severity than the control group. In the metabolic pattern, cognitive disorders are also present. In the musculoskeletal-metabolic pattern, almost every man has osteoarthritis, while additional comorbidity is also common. The two other networks showed a higher clinical complexity in additional comorbidity and a higher likelihood of COVID-19 severity. Acute cerebrovascular and cardiovascular diseases, neurological disorders, and cognitive problems were amongst the main coexisting conditions in the cerebro-cardiovascular-metabolic pattern. Cardiovascular and respiratory diseases were significant comorbidity in the cardiovascular-respiratory-metabolic pattern, the multimorbidity network with the highest likelihood of hospitalization and mortality in men (70.5% higher than the control group).

### 3.2. Multimorbidity Profiles in Women

We observed six multimorbidity patterns in young and middle-aged women. Menstrual disorders were a common finding in all profiles and the main comorbidity in the menstrual pattern. We observed a pattern of women with anxiety and another with a headache. In the respiratory pattern, individuals with respiratory conditions and asthma frequently had eye infections. Another common multimorbidity network was the profile with mental health problems. The cardiovascular-musculoskeletal-metabolic pattern was the clinical profile with the highest likelihood of hospitalization and mortality in women 15–64 years of age (6.8% higher than the control group).

We observed five multimorbidity profiles in the elderly population 65–79 years of age. Arterial hypertension, dyslipidemia, osteoarthritis, and spine/back problems are present in all multimorbidity networks in this age group. Their coexistence has been associated with a higher likelihood of COVID-19 severity than the control group, with differences related to their prevalence and additional comorbidity. The clinical profile of the metabolic-musculoskeletal pattern corresponds to the coexistence of these four conditions. The eye disorders-musculoskeletal-metabolic pattern also includes eye disorders and the cardiometabolic pattern, diabetes, and obesity. The mental health-metabolic pattern has a more complex clinical profile with 14 diseases on average, including metabolic conditions, mental health problems, cognitive, and sensory organ disorders. The cardiovascular-respiratory-metabolic pattern was the network with the highest likelihood of hospitalization and mortality (43.5% higher than the control group) and the highest mean number of chronic conditions (17.5 diseases) in this age group.

In the oldest-old population, we observed four multimorbidity patterns. Arterial hypertension, dyslipidemia, diabetes mellitus, osteoarthritis, back problems, and cognitive disorders are present in all multimorbidity networks in this age group. Their coexistence has been associated with a higher likelihood of COVID-19 severity than the control group. The clinical profile of the degenerative pattern corresponds to the coexistence of these six conditions. In the cataract-degenerative profile, almost all women also have a cataract, and some of them, osteoporosis. Mental health problems were common comorbidity in the mental health pattern. Cardiovascular, kidney, and respiratory diseases were significant comorbidity in the cardiovascular-metabolic pattern, the multimorbidity profile with the highest likelihood of hospitalization and mortality in oldest-old women (34.6% higher than the control group).

## 4. Discussion

This work revealed that people with multiple chronic conditions are more likely to suffer a more severe SARS-CoV-2 infection than people without multimorbidity in terms of hospitalization and mortality. Our findings strongly support the hypothesis that multimorbidity is a risk factor of poorer outcomes in all ages and both sexes and that this risk seems to be also determined by the baseline clinical profile of the infected individuals [13]. Network science allowed us to apply sophisticated techniques and explore in what grade different multimorbidity profiles are associated with hospitalization and mortality. We saw that many clinical profiles included diseases that had not been previously reported as risk factors of COVID-19 severity (e.g., menstrual disorders), or for which there were discrepancies between different studies (e.g., hypertension [14,37,38], or psychiatric disorders [39,40]), suggesting that various yet to be discovered factors could be present, such as disease–disease interactions, common etiopathogenic pathways, drug–disease, and drug–drug interactions, and others. 

Advanced age and male sex have been extensively reported as the main risk factors of adverse outcomes in COVID-19 [41,42,43,44,45]. One in two oldest-old men and one in three oldest-old women had a severe infection in our study. Although most infected individuals were young and middle-aged adults, 5% were admitted to hospitals or died. This highlights the importance of considering all age groups when searching for severity risk factors. We saw that multimorbidity was a widespread finding in all ages and sexes. Conditions that had been previously associated with COVID-19 adverse outcomes [12,13,14,46,47,48], such as cardiovascular diseases, diabetes, obstructive pulmonary diseases, chronic renal failure, and neoplasms, were present in various multimorbidity networks, sometimes in clinically unexpected combinations. The clinical component of each multimorbidity profile is one factor that determines the risk of COVID-19 severity. We found that people with multimorbidity could have up to 70% higher risk of hospitalization and mortality, as is the case of the oldest-old men with a high morbidity burden that includes cardiovascular and kidney diseases.

Metabolic diseases were the main comorbidity in various multimorbidity networks, especially in those with the highest risk of COVID-19 severity. Many observational works have studied the risk of each condition separately and reported that dyslipidemia, diabetes, and obesity are associated with poorer prognoses in SARS-CoV-2 infected patients [49,50,51,52]. It has been suggested that even prediabetes and increased glycemic status could act as risk factors of COVID-19 severity [53,54]. Although there is significant inconsistency in the literature regarding hypertension [14,37,38], our findings showed that arterial hypertension was present in various high-risk multimorbidity networks, and it was frequently coexisting with diabetes, dyslipidemia, and obesity. The existence of systematic associations between these conditions is well-documented in the literature, showing that they tend to cluster in a metabolic pattern, and consequently, with the potential to develop metabolic syndrome if specific criteria are met [18]. The coexistence of these conditions, although preventable, is challenging in clinical practice because it constitutes a risk factor of cardiovascular comorbidity.

In the elderly population, we saw that multimorbidity networks with metabolic and cardiovascular diseases had the highest risk of COVID-19 hospitalization and mortality. Cardiovascular comorbidity has been associated with poorer prognosis in SARS-CoV-2 infected individuals, and many authors have suggested that people with preexisting cardiovascular diseases may need special attention [12,13,14,55]. In the general population, especially in the elderly, a cardiorespiratory pattern has been identified; the association between cardiovascular and pulmonary diseases is well known in the literature and of great importance in clinical practice [18,56]. In this study, we saw that patients with cardiovascular, metabolic, and respiratory comorbidity are more likely to suffer a more severe course of COVID-19. The multimorbidity profiles with the coexistence of these conditions and additional comorbidities, such as kidney disease and musculoskeletal conditions, were the clinical profiles with the highest likelihood of hospitalization and mortality. An interesting fact that should be considered and further studied, not only in the context of COVID-19, is that most of the chronic conditions that compose the identified clinical profiles could predispose to chronic systemic inflammation [18] and thus to a higher risk of coagulopathies and thromboembolic events [57,58]. The high risk of advanced cardiometabolic profiles, probably related to chronic systemic inflammation, is consistent with our previous study on multimorbidity profiles associated with COVID-19 severity from primary care settings in the northern Spanish region of Aragón [23].

Mental health patterns were also among the profiles with the highest severity risk in Andalusia, especially in women. This result is also consistent with our previous study in Aragón [23] and with a recent systematic review and meta-analysis [39]. However, a Mendelian Randomization study found that the association between psychiatric conditions and COVID-19 severity could be related to statistical models not adequately analyzing the body mass index as a continuous covariate [40]. The role of mental health problems in severity risk is not clear. It is thought that mental diseases could have an impact on protective factors for COVID-19 perceived risk [59]. Further studies are needed to clarify the potential role and mechanisms of mental health problems in COVID-19 severity.

Our findings strongly support that it should be highly recommended to implement personalized approaches to chronic patients with multimorbidity infected with SARS-CoV-2, especially in the elderly population with high morbidity burden. Similar conclusions have been reported in a large-scale epidemiologic study in middle-aged adults in the United Kingdom. Considering the most common chronic conditions, the authors found that multimorbidity was independently associated with a nearly two times higher likelihood of COVID-19 severity [60]. The authors reported that the risk of COVID-19 hospitalization and mortality varies amongst the different combinations of diseases, with the highest risk observed for individuals with cardiovascular disease and diabetes.

The main strength of this research is the large-scale population-based nature of the study, which included all individuals with laboratory-confirmed COVID-19 infection in the reference population. Furthermore, we exhaustively analyzed virtually all chronic conditions registered in primary sources of information (i.e., EHRs) from both primary and hospital care, and not only those most prevalent, relevant, or self-reported by the patients. Another strength is the study’s innovative approach to detect groups of infected patients based on their multimorbidity using network analysis. Network analysis and the approach applied in this study could facilitate the replicability and automation of the analysis compared with other methods such as classical cluster approaches that also allow analyzing patients and their impact but with frequent computational limitations (e.g., agglomerative hierarchical clustering) and higher grade of subjectivity (e.g., k-means algorithm) [19,23]. However, this approach was also not free of subjectivity regarding the selection of the diseases to be part of each of the multimorbidity patterns (based on thresholds proposed in previous studies) and their clinical interpretation. Another important limitation is that our database only included information on all-cause mortality and not on the cause of death, so we could not assess the direct association between COVID-19 infection and death. On the other hand, some variables that were not available in our study could have been relevant in the interpretation of the results, such as socioeconomic variables, genetics, laboratory tests, inpatient treatments, and lifestyle habits (e.g., smoking, drinking, or physical activities), amongst others.

## 5. Conclusions

In this large-scale epidemiological study, we were able to identify multimorbidity profiles in the SARS-CoV-2 infected population and explore their association with hospitalization and mortality. By applying advanced statistical techniques, we saw that the clinical component of each multimorbidity profile determines the risk of COVID-19 severity. We found that multimorbidity is a risk factor for COVID-19 severity and that this risk increases with the morbidity burden. Men and women with advanced cardiometabolic profiles presented the highest severity risk, whereas women with mental health patterns were also among the profiles associated with higher infection severity. Our findings strongly recommend the implementation of person-centered approaches to all chronic patients with multimorbidity, especially in those with high morbidity burdens, in order to avoid preventable consequences on health during the pandemic.

## Figures and Tables

**Table 1:** Demographic and clinical characteristics of individuals aged 15 years or older with COVID-19 infection in the Andalusian Population Health Database.

Characteristics	Total (N = 166,242)	Men (N = 73,031)	Women (N = 93,211)
**Mean age** (SD)	47.4 (19.6)	47.0 (19.2)	47.8 (19.9)
**Age interval** (N, %)			
15–64 years	133,538 (80.3%)	59,056 (80.9%)	74,482 (79.9%)
65–79 years	20,186 (12.1%)	9638 (13.2%)	10,548 (11.3%)
≥80 years	12,518 (7.5%)	4337 (5.9%)	8181 (8.8%)
**Mean number of chronic diseases** (SD)	5.9 (4.7)	5.3 (4.6)	6.4 (4.8)
**Multimorbidity †** (N, %)			
Total	145,070 (87.3%)	61,109 (83.4%)	83,961 (90.1%)
15–64 years	112,814 (84.5%)	47,362 (80.2%)	65,452 (87.9%)
65–79 years	19,822 (98.2%)	9442 (98.0%)	10,380 (98.4%)
≥80 years	12,434 (99.3%)	4305 (99.2%)	8129 (99.4%)
**Outcomes**			
**Hospitalization** (N, %)	15,717 (9.5%)	8523 (11.7%)	7194 (7.7%)
**30-day mortality** (N, %)	3821 (2.3%)	1997 (2.7%)	1824 (2.0%)
**Severity ††** (N, %)			
Total	16,635 (10.0%)	8895 (12.2%)	7740 (8.3%)
15–64 years	6684 (5.0%)	3831 (6.5%)	2853 (3.8%)
65–79 years	5026 (24.9%)	2940 (30.5%)	2086 (19.7%)
≥80 years	4925 (39.3%)	2124 (49.0%)	2801 (34.2%)

† Multimorbidity, defined as the simultaneous coexistence of two or more chronic diseases, excluding COVID-19; SD—standard deviation; †† Severity, defined as a composite outcome based on the need for hospital admission (including in Intensive Care Units) or 30-day all-cause mortality.

**Table 2:** Denomination and prevalence of multimorbidity patterns found in COVID-19 patients based on sex and age and their age-adjusted odds ratios (aORs) of infection severity compared with chronic patients without multimorbidity.

	15–64 Years Pattern Name (N, Prevalence)	Severity aOR (95% CI) Mean # Diseases (SD)	65–79 Years Pattern Name (N, Prevalence)	Severity aOR (95% CI) Mean # Diseases (SD)	≥80 Years Pattern Name (N, Prevalence)	Severity aOR (95% CI) Mean # Diseases (SD)
**Men**	Cardiovascular-metabolic (11,273, 19.1%)	1.14 (1.13–1.14) 7.28 (4.32)	Cardiovascular-respiratory-metabolic (3020, 31.4%)	1.43 (1.34–1.53) 15.55 (4.72)	Cardiovascular-respiratory-metabolic (1241, 28.7%)	1.71 (1.44–2.02) 16.77 (4.77)
	Neurologic-mental health (7545, 12.8%)	1.05 (1.04–1.06) 3.16 (1.72)	Prostatic-metabolic (1486, 15.4%)	1.20 (1.12–1.28) 7.28 (2.86)	Cerebro-cardiovascular metabolic (606, 14.0%)	1.58 (1.33–1.89) 15.23 (4.37)
	Dyslipidemia (6239, 10.6%)	1.05 (1.04–1.06) 4.27 (2.08)	Metabolic (2599, 27.0%)	1.16 (1.08–1.24) 5.74 (2.56)	Musculoskeletal-metabolic (1088, 25.2%)	1.44 (1.21–1.72) 10.59 (3.69)
	Mental health (7383, 12.5%)	1.03 (1.02–1.04) 4.87 (2.80)	Musculoskeletal-metabolic (2320, 24.1%)	1.14 (1.06–1.22) 8.81 (3.30)	Metabolic (1354, 31.3%)	1.43 (1.20–1.70) 8.08 (3.32)
	Respiratory (6154, 10.4%)	1.01 (1.00–1.02) 3.58 (1.71)				
	Eye disorders-respiratory (4178, 7.1%)	1.01 (0.99–1.02) 5.01 (2.36)				
	Sensory organ disorders (4576, 7.8%)	1.01 (0.99–1.02) 4.08 (2.10)				
	Control group (11,694, 19.8%)		Control group (196, 2.0%)		Control group (32, 0.7%)	
	**15–64 years** **Pattern Name** **(N, prevalence)**	**Severity aOR (95% CI)** **Mean # Diseases (SD)**	**65–79 Years** **Pattern Name** **(N, prevalence)**	**Severity aOR (95% CI)** **Mean # Diseases (SD)**	**≥80 years** **Pattern Name** **(N, Prevalence)**	**Severity aOR (95% CI)** **Mean # Diseases (SD)**
**Women**	Cardiovascular-musculoskeletal-metabolic (12,012, 16.1%)	1.07 (1.06–1.07) 6.88 (3.72)	Cardiovascular-respiratory-metabolic (753, 7.1%)	1.44 (1.34–1.54) 17.58 (5.64)	Cardiovascular-metabolic (2371, 29.0%)	1.35 (1.18-1.53) 16.46 (5.10)
	Mental health (13,903, 18.7%)	1.04 (1.04–1.05) 8.36 (4.36)	Mental health-metabolic (3073, 29.2%)	1.17 (1.09–1.24) 14.17 (5.15)	Mental health (1881, 23.0%)	1.21 (1.07-1.38) 14.74 (4.50)
	Headache-menstrual (7339, 9.9%)	1.01 (1.01–1.02) 5.11 (2.35)	Cardiometabolic (2105, 20.0%)	1.16 (1.09–1.24) 9.80 (3.83)	Degenerative (2014, 24.6%)	1.17 (1.03–1.34) 7.67 (3.22)
	Menstrual (13,760, 18.5%)	1.01 (1.01–1.02) 3.38 (1.60)	Eye disorders-musculoskeletal-metabolic—(1302, 12.4%)	1.08 (1.01–1.15) 9.61 (3.67)	Cataract-degenerative (1855, 22.7%)	1.15 (1.01–1.31) 9.81 (3.18)
	Dysphoric-menstrual (4798, 6.4%)	1.01 (1.00–1.01) 4.15 (1.91)	Metabolic-musculoskeletal (3134, 29.7%)	1.07 (1.00–1.14) 6.56 (3.02)		
	Respiratory (13,626, 18.3%)	1.01 (1.00–1.01) 5.20 (2.44)				
	Control group (9030, 12.1%)		Control group (168, 1.6%)		Control group (52, 0.6%)	

Mean # diseases (SD)—mean number of chronic diseases (standard deviation); Control group—chronic patients without multimorbidity; CI—confidence interval.

## Data Availability

The data used in this study cannot be publicly shared because of restrictions imposed by the Andalusian Health Service (SAS) and asserted by the Clinical Research Ethics Committee of Andalusia (CCEIBA). The authors who accessed the data belong to the Andalusian Health Service or received permission from SAS to utilize the data for this specific study, thus implying its exclusive use by the researchers appearing in the project protocol approved by CCEIBA. The authors can establish future collaborations with other groups based on the same data. However, each new project based on these data has to be previously submitted to the CCEIBA to obtain the respective mandatory approval. Potential collaborations should be addressed to the Research Coordinator, Román Villegas, at romanp.villegas@juntadeandalucia.es.

## Supplementary Materials

The following supporting information can be downloaded at: https://www.mdpi.com/article/10.3390/ijerph19073808/s1.
